# Supplementary material for: 2′-Fucosyllactose helps butyrate producers outgrow competitors in infant gut microbiota simulations
Source: iScience. 2024 Feb 3;27(3):109085. doi: 10.1016/j.isci.2024.109085 (PMC10877688; doi:10.1016/j.isci.2024.109085)
Supplement: Document S1. Figures S1–S3 [file mmc1.pdf]

iScience, Volume *27*

Supplemental information

2′-Fucosyllactose helps butyrate producers outgrow competitors in infant gut microbiota simulations

David M. Versluis, Ruud Schoemaker, Ellen Looijesteijn, Jan M.W. Geurts, and Roeland M.H. Merks

Supplemental information

S1 Table, related to Figure 1.

Table of changed or deleted reactions and annotations.csv

A table of changes made to the AGORA models as a .csv file.

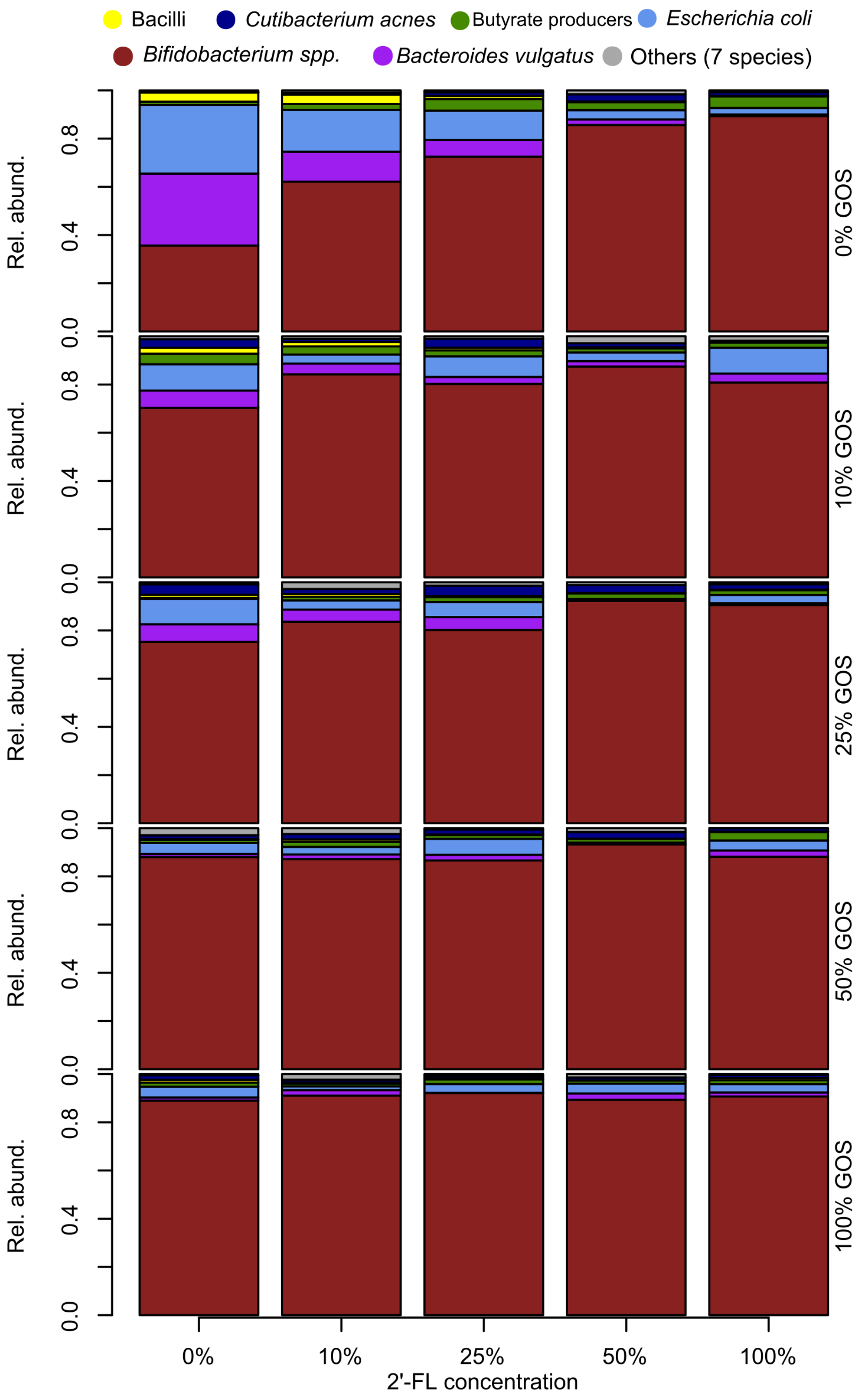

Figure S1.

Relative abundance of bacterial species at the end of 21 days with varying inputs of 2'-FL and GOS compared to the fixed input amount of lactose, related to Figure 2. n=30 for each condition, each simulation is weighed equally.

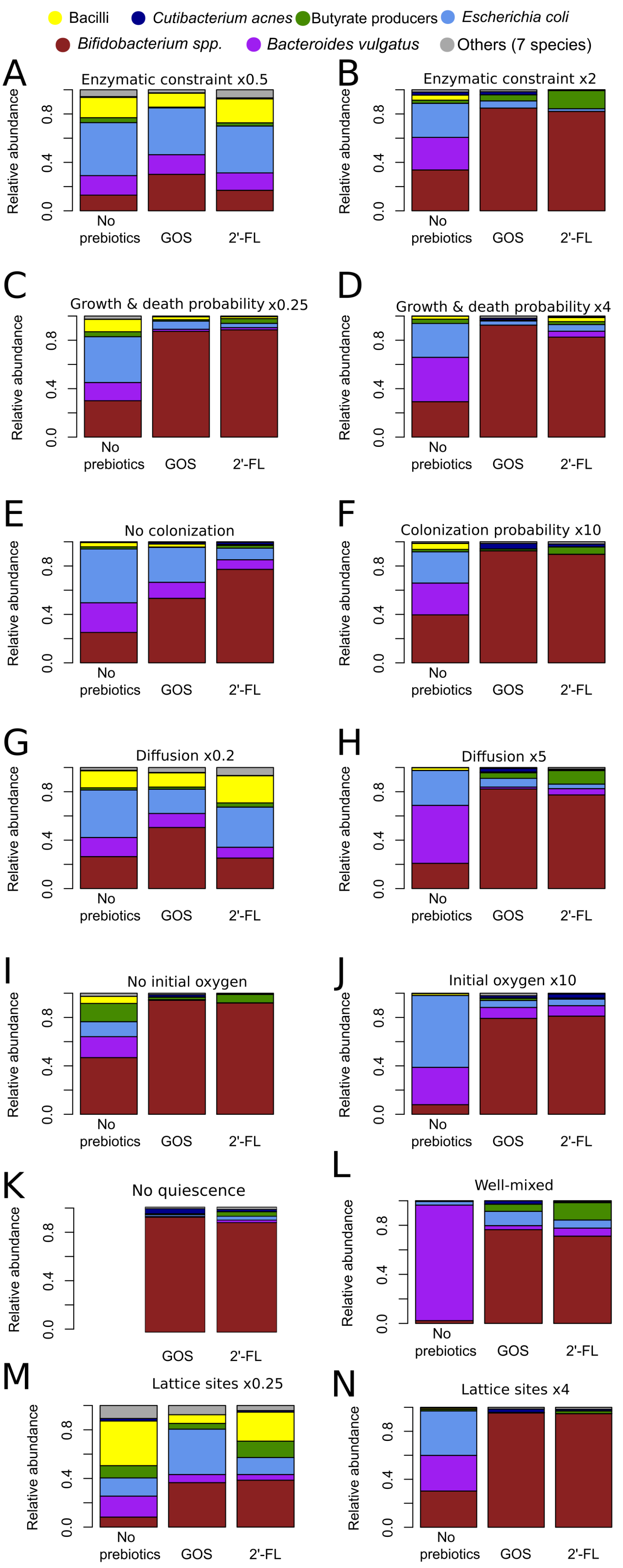

Bacilli
Cutibacterium acnes
Butyrate producers
Escherichia coli
Bifidobacterium spp.
Bacteroides vulgatus
Others (7 species)
A
Enzymatic constraint x0.5
B
Enzymatic constraint x2
C
Growth & death probability x0.25
D
Growth & death probability x4
E
No colonization
F
Colonization probability x10
G
Diffusion x0.2
H
Diffusion x5
I
No initial oxygen
J
Initial oxygen x10
K
No quiescence
L
Well-mixed
M
Lattice sites x0.25
N
Lattice sites x4
Relative abundance
0.0
0.4
0.8
No prebiotics
GOS
2'-FL

Figure S2.

(A to N) Relative abundance of bacterial species in the conditions with no prebiotics, with GOS, or with 2'-FL at the end of 21 days, related to Figure 2., with the following alteration from the baseline of Fig. 2A: (A) Enzymatic constraint loosened by a factor of 2, to 4 µmol flux per timestep per $1 \cdot 10^{10}$ population (B) Enzymatic constrained tightened by a factor of 2, to 1 µmol flux per timestep per $1 \cdot 10^{10}$ population (C) Growth decreased by a factor of 4, by increasing the ATP to grow one bacterium to $4 \cdot 10^{-15}$, with the death probability decreased to 0.001875 per population per timestep. (D) Growth increased by a factor of 10 by decreasing the ATP to grow one bacterium to $2.5 \cdot 10^{-16}$, with the death probability increased to 0.03 per population per timestep (E) Colonisation removed by setting the probability for new populations to be placed after initialization to 0 (F) Colonisation increased by x10 by setting the probability per empty lattice to acquire a new population to 0.0005 per timestep (G) Diffusion of both metabolites and bacteria decreased by a factor of 5 to $1.26 \cdot 10^{-6}$ cm^2/s (H) Diffusion of both metabolites and bacteria increased by a factor of 5 to $3.15 \cdot 10^{-5}$ cm^2/s (I) No initial presence of oxygen (J) Initial oxygen increased to 1 µmol per lattice site (K) Quiescence disabled (L) Well-mixed conditions (M) Number of lattice sites decreased by a factor of 4 (N) Number of lattice sites increased by a factor of 4

For each: n=30 for each condition, each simulation is weighed equally.

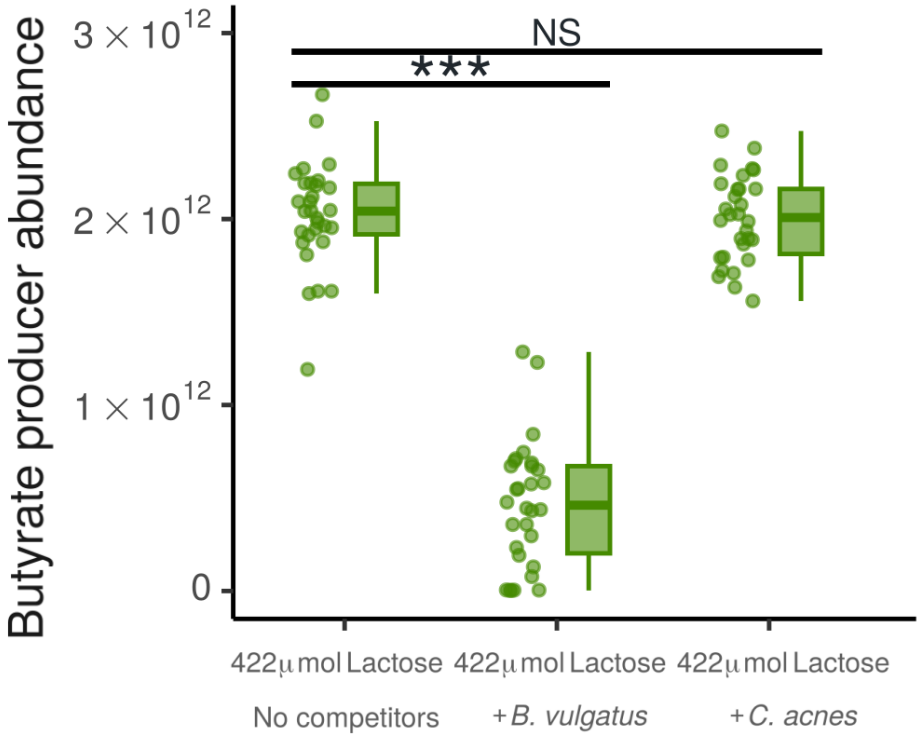

Figure S3.

Abundance of butyrate producing bacteria at the end of 21 days with 422 μmol of lactose per three hours and without prebiotics, either without competitors (only *Bifidobacterium* and butyrate producing bacteria), with addition of *B. vulgatus*, or with addition of *C. acnes*, related to Figure 3. n=30 for each condition. Each simulation is represented by one dot.

NS: Not significant, *: $p<0.05$, **:$p<0.01$, ***:$p<0.001$
